# Antibacterial Activity of Metabolites of Endophytic *Bacillus subtilis* Isolated From Selected Ethiopian Medicinal Plants

**DOI:** 10.1155/ijm/7403296

**Published:** 2025-10-06

**Authors:** Tsedale Tasew, Dagim Jirata Birri, Fitsum Tigu, Dereje Beyene, Tegenu Gelana, Ketema Tolossa, Balako Gumi, Tigist Getachew, Iris Bertani, Vittorio Venturi, Dejene Guta, Asnake Desalegn

**Affiliations:** ^1^Department of Microbial Sciences and Genetics, College of Natural and Computational Sciences, Addis Ababa University, Addis Ababa, Ethiopia; ^2^College of Natural and Computational Sciences, Department of Biology, Kotebe University of Teachers Education (KUTE), Addis Ababa, Ethiopia; ^3^Aklilu Lemma Institute of Pathobiology, Addis Ababa University, Addis Ababa, Ethiopia; ^4^Bio and Emerging Technology Institute (BETin), Addis Ababa, Ethiopia; ^5^International Centers for Genetic Engineering and Biotechnology, Trieste, Italy

**Keywords:** antibacterial activity, *Bacillus subtilis*, endophyte, medicinal plants, minimum bactericidal concentration, minimum inhibitory concentration

## Abstract

Antimicrobial resistance remains a global concern, and there has been sustained exploration of natural products with therapeutic effects. Endophytes are among the potential microorganisms considered as the treasure chest for bioactive secondary metabolites with therapeutic effects. The current study was aimed at evaluating the antibacterial activity of endophytic *Bacillus subtilis* isolated from selected Ethiopian medicinal plants (*Kalanchoe petitiana*, *Artemisia abyssinica*, *Rumex abyssinicus*, and *Clematis longicauda*). Collection of the plant samples, endophyte isolation, metabolite production, and antimicrobial activities were conducted following standard protocols. The isolates were further identified at the molecular level using 16S rRNA gene sequence. The endophytic crude extract demonstrated inhibition zones (millimeter) ranging from 6.00 ± 0.00 against *Pseudomonas aeruginosa* to 22.00 ± 0.71 against *Salmonella* Typhimurium. In general, *S.* Typhimurium was the most sensitive, followed by *Escherichia coli*, but *P. aeruginosa* was the least sensitive to the extracts. The extract from isolate En6 produced the largest inhibition zone against *Staphylococcus aureus*, but the extract from Ch1 produced pronounced inhibition against *E. coli*, *S.* Typhimurium, and *P. aeruginosa*. The lowest minimum inhibitory concentration and minimum bactericidal concentration values ($\bar{x}\pm \text{SD}$ in milligram/milliliter) of 0.025 ± 0.01 and 0.98 ± 0.01, respectively, were recorded against *S.* Typhimurium by the extract from isolate Ch1. Molecular characterization confirmed that the isolates were members of *B. subtilis.* This is the first report of the isolation of endophytes with antibacterial activities from the Ethiopian medicinal plants. The strong inhibitory activity of the metabolites against the selected test bacteria shows the potential of the endophytic metabolites for use as antibacterial agents with further studies.

## 1. Introduction

Medicinal plants have long been used to treat ailments worldwide for thousands of years and still remain as important as a primary healthcare resource for the majority of the world's population and are also used as raw materials for various drug developments [1]. Indeed, traditionally used herbal medicines have been behind the success of many drug discoveries. It is also thought that these plants can be used as remedies against drug-resistant pathogens [1]. Bioactive compounds derived from medicinal plants play an important inhibitory role against various human pathogens [2–4].

Vascular plants harbor endophytic microorganisms that reside within their tissues without causing apparent harm [5]. Endophytes that live in plants have mutual associations with the host plants [6]. The majority of endophytic bacteria belong to the phyla Actinobacteria, Proteobacteria, and Firmicutes and are well known for their ability to produce antimicrobial compounds [7]. Different species of bacteria have been reported to produce antimicrobial agents against human pathogens [8, 9]. However, *Bacillus* species are among the most frequently isolated bacteria from endophytic sources with the potential to produce antimicrobial compounds. *Bacillus* species are Gram-positive, endospore-forming members of the phylum Firmicutes and are mostly found in soil as well as in association with plants. The members of the *Bacillus subtilis* species have been reported to produce bioactive secondary metabolites with significant activity against plant pathogens [10]. Other studies also demonstrated the role of *B. subtilis* in the control of plant pathogens, plant growth promotion, and other plant beneficial traits [11–15].

The antimicrobial activities of *B. subtilis* against human pathogens were reported by [16], where the bacteria produced broad-spectrum antimicrobial peptides. In another report, endophytic *B. subtilis* from medicinal plants demonstrated antibacterial activity particularly against *Staphylococcus aureus* and *Escherichia coli* [17]. The bacteria possessed biosynthetic genes that enabled them to produce antibiotics such as bacitracin, subtilosin, and surfactin that act by inhibiting the cell wall synthesis and disrupting membrane integrity [11].

Even though there are several studies on the role of different *Bacillus* species in plant growth promotion, there are limited studies on the role of these bacterial species on human pathogenic microorganisms, and there has been no report in Ethiopia regarding the antibacterial activities of endophytic *B. subtilis* isolated from medicinal plants. This study was aimed at evaluating the antibacterial activity of selected endophytic *B. subtilis* species isolated from four Ethiopian medicinal plants: *Kalanchoe petitiana*, *Artemisia abyssinica*, *Rumex abyssinicus*, and *Clematis longicauda*.

## 2. Materials and Methods

### 2.1. Description of Sampling Sites

The study area was Tenlole Kebele (the smallest administrative unit), Berhena Aleltu District, Northern Shewa Zone, Oromia Regional State. The sampling site is located 30 km from the Ethiopia's capital city, Addis Ababa. The area is characterized by moderate temperature conditions. The medicinal plants have been used by the local community for the treatment of sore throat and inflammation (*K. petitiana*), common cold and upper respiratory infections (*A. abyssinica*), chronic cough (*R. abyssinicus*), and dermal infection, cutaneous wounds, and warts (*C. longicauda*).

### 2.2. Plant Sample Collection

Young and healthy plant samples were collected and separately placed in sterile polyethylene bags in duplicate and then transported to the Applied Microbiology Laboratory, Department of Microbial Sciences and Genetics, Addis Ababa University, using an ice box according to the method used in [18]. The plant specimens were identified by Mr. Melaku Wendafrash, who is an expert from the Department of Plant Biology and Biodiversity Management, College of Natural and Computational Sciences, Addis Ababa University, and stored with herbarium voucher numbers T.T.003 (*K. petitiana* A. Rich.), T.T.004 (*C. longicauda* Perr. & Guill), T.T.005 (*R. abyssinicus* Tocq.), and T.T.006 (*A. abyssinica* Sch. Bip. Ex A. Rich) at the herbarium of the Addis Ababa University, College of Natural and Computational Sciences.

### 2.3. Pretreatment and Surface Sterilization of the Plant Samples

Plant sample pretreatment and surface sterilization were done following the method reported in [19], with some modifications. The plant samples were washed under tap water for 3 min, then with sterile distilled water, and soaked in 70% ethanol for 1 min, followed by 5% sodium hypochlorite (Sigma-Aldrich, Germany, CAS Number 7681-52-9) for 5 min and 90% ethanol for 1 min. Finally, the samples were rinsed five times using sterile distilled water, and the last rinse was used as a control for the sterility test.

### 2.4. Culturing and Isolating Endophytic Bacteria

**Figure 1:**
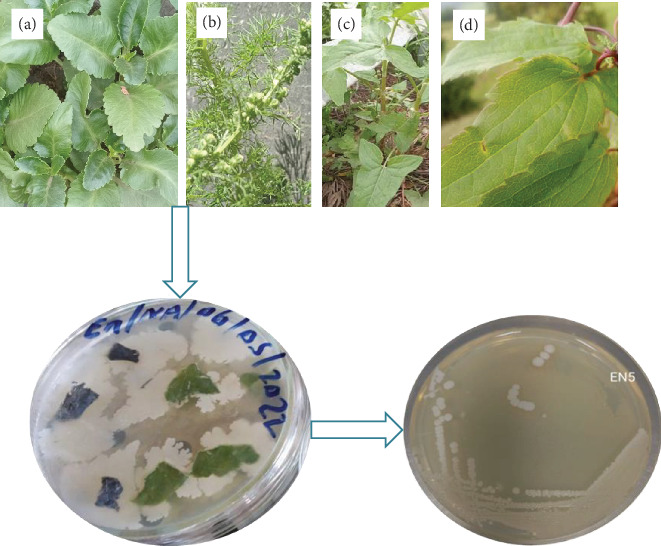
Isolation and purification of endophytic bacteria from the host plants. (a) *K. petitiana*. (b) *A. abyssinica*. (c) *R. abyssinicus*. (d) *C. longicauda*.

Culturing bacterial endophytes was done using the method in [19], with some modifications. First, surface-sterilized leaf and roots were kept in a safety cabinet until the surface water was removed, and then, the parts were cut into small pieces (about 5 mm). The pieces were stamped onto the surfaces of sterilized nutrient agar (NA) (Millipore, Germany) medium supplemented with fluconazole (10 *μ*g/mL) to avoid fungal growth. Simultaneously, 30 *μ*L of the sterile distilled water used as the final rinse was also inoculated onto the sterilized NA plates for a sterility test. All plates were then sealed with parafilm and incubated at 28°C for 48 h. After proper incubation, colonies arising from the cut ends of the plant parts were separately transferred into nutrient broth and incubated. Then, a sterile inoculating loop was immersed into the culture broth and streaked onto presterilized NA medium and incubated. This process was repeated until a pure colony of the endophyte was obtained, and pure cultures obtained were kept at −4°C on a NA slant until further analysis [20]. The plants used in this study and isolation of endophytes are indicated in Figure 1.

### 2.5. Preliminary Screening of Endophytes for Their Antibacterial Activity

The preliminary antibacterial screening assay was done using the method in [21]. The endophytic bacterial broth fermented for 5 days at 28°C under submerged fermentation conditions was centrifuged using an Eppendorf centrifuge (Centrifuge 5418 R-Germany) for 30 min at 7500 rpm and 4°C. The supernatant was then transferred into a sterile Eppendorf tube. A positive control, ciprofloxacin (10 *μ*g/mL), was prepared in sterile dH_2_O, and the sterile dH_2_O was used as a negative control. Standard strains of *E. coli* (ATCC25922), *Pseudomonas aeruginosa* (ATCC27853), *S. aureus* (ATCC25923), and *Salmonella* Typhimurium (ATCC12228) were obtained from the Ethiopian Public Health Institute (EPHI). For adjusting the bacterial inoculum level, 0.5 McFarland standard, equivalent to a culture concentration of about 10^8^ CFU/mL, was prepared. The suspensions of the test bacteria were prepared using a 24-h culture in physiological saline, and a comparison with the McFarland standard was made against white paper with black strips. The antimicrobial activity of the endophytic metabolite was determined using the agar well diffusion method [21]. In the assay, 6-mm wells were punched onto the preinoculated Mueller–Hinton agar (MHA) plates using a sterile cork borer. The wells were filled with 30 *μ*L of the supernatants of the respective endophytic bacterial culture broth, the positive and negative controls. After incubation at 37°C for 24 h, inhibition zones surrounding the wells were measured (millimeter) using a caliper. The assays were done in triplicate.

### 2.6. Preparation of the Crude Extracts of the Endophytic *B. subtilis*

The preparation of the crude extract of the endophytic bacteria was done following the method reported in [22]. Sterile nutrient broth (500 mL) amended with antifungal agents was prepared in a 1000-mL Erlenmeyer flask (Millipore, Germany, Catalogue Number 105443) and inoculated with 200 *μ*L of 24-h culture of the respective endophytic bacteria. The cultures were then incubated at 28°C for 7 days on a rotary shaker at 130 rpm. After incubation, an equal volume of methanol (Sigma-Aldrich, Germany, CAS Number 67-56-1) was added to each culture broth and was incubated for 24 h at 4°C. The culture suspensions were then centrifuged using a Gallenkamp angle head centrifuge (England, CFB-700-010C) at 5000 rpm for 30 min. The resulting supernatants were then filtered with Whatman No. 1 filter paper (Fisher Scientific, United Kingdom, Product Code 10424081). The methanol was evaporated from the filtrate using a rotary evaporator (PHOENIX, Germany, RE-100D) at 40°C and 90 rpm. The remaining aqueous solution was finally freeze-dried using a lyophilizer (CHRIST/D-37520-Germany), and the resulting crude metabolites were stored at −4°C for further work.

#### 2.6.1. Bioassay-Guided Fractionation of Crude Metabolites

**Figure 2:**
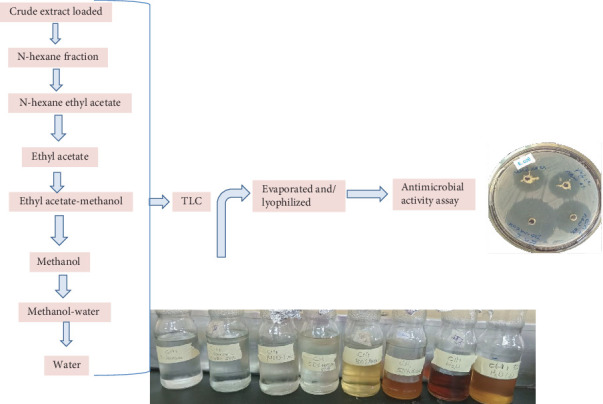
Overall procedures in the bioassay-guided open column fractionation of the crude metabolite of endophytic *B. subtilis.*

Open column fractionation of crude endophytic *B. subtilis* metabolites was done using bioassay-guided fractionation with different solvent systems in accordance with the procedures in [23], with some modifications. The first fractionating solvent used was N-hexane, followed by n-hexane-ethyl-acetate (EtOAc). Each fraction was loaded onto TLC and visualized under UV light at longer wavelength (366 nm) and shorter wavelength (254 nm). Since no major visible compound was observed up to the n-hexane-EtOAc 1:1 solvent system, fractions below the polarity of n-hexane-EtOAc 1:1 were discarded (Figure 2). In addition, no major antimicrobial active compound-containing fraction was found up to the EtOAc-MeOH (methanol) 1:1 solvent system; for this reason, fractions of all the samples were collected starting from the solvent system EtOAc-MeOH 1:1 toward the polar water based on bioassay results.

### 2.7. MIC and MBC Assays for Fractions of Endophytic *B. subtilis* Metabolites

The MIC and the MBC of the selected fractions of the endophytic *B. subtilis* metabolites were done by broth macrodilution standard method (CLSI M07-A8), using Mueller–Hinton Broth (Millipore, Germany). The test was done against the human pathogenic bacteria used in the preliminary assays, *S. aureus* (ATCC25923), *E. coli* (ATCC25922), *S.* Typhimurium (ATCC12228), and *P. aeruginosa* (ATCC27853). The 0.5 McFarland standard of each standard test bacterial suspension was diluted into 1:150 to obtain about 5 × 10^6^ CFU/mL and followed by 1:2 dilutions with a given sample, where 1 mL of the 5 × 10^6^ CFU/mL suspension was added to each of the twofold broth dilution test tubes. One milliliter of sample was then added to the first higher concentration test tube with defined bacterial suspension and mixed well, followed by consecutive serial dilutions to the next concentrations. Finally, 1 mL of the mixture was discarded from the last tube to bring the volumes to equal level. The same volume (1 mL) of sterile broth was used as a positive control, and a tube with 1 mL of bacterial suspension with no extract was also used as a negative control. All test tubes were then incubated for 24 h at 37°C. The smallest concentration with no turbidity was considered as the MIC of the respective fraction. Determinations of the MBC of the endophytic extracts were performed by subculturing the broth without visible growth on MHA media at 37°C for 24 h. The MBC was defined as the lowest concentration of the subcultured plates that did not show any visible growth of bacteria.

### 2.8. Molecular Identification of the Isolates

#### 2.8.1. DNA Extraction

For 16S rRNA sequence, approximately 2–4 mL aliquots of active log-phase 24–48 h bacterial culture were used. Bacterial cells were harvested by centrifugation (at 5000 rpm for 5 min). The cell pellet was then washed three times using 1 mL NaCl-EDTA (30 mM NaCl, 2 mM EDTA, pH 8.0). DNA was extracted following phenol–chloroform and isoamyl alcohol (25:24:1) protocol [24], with some modifications. The extracted DNA pellet was washed with freshly prepared 70% ethanol and air-dried. The resulting DNA was eluted in 50 *μ*L of sterile distilled water. The quality and quantity of the resulting DNA (integrity of DNA) were measured using gel electrophoresis (0.8% agarose gel) and NanoDrop (OD260/OD280 ratio) using TM One C: Microvolume UV-Vis Spectrophotometer from Thermo Scientific (United States). The DNA with appropriate quality was stored frozen at −20°C for further use.

#### 2.8.2. Amplification of Extracted DNA Using PCR Primer

The extracted DNA samples were used as templates for PCR amplification. 27F as forward primer = 5⁣′ AGA GTT TGA TCC TGG CTC AG 3⁣′ and rP2Runi as reverse primer = 5⁣′ ACG GCT ACC TTG TTA GGA CTT 3⁣′ (Eurofins Genomics, Germany) were general primers obtained from the data bank and used for PCR. The PCR master mix used was 27.75 *μ*L sterile nuclease-free water, 10 *μ*L of Go buffer 5X, 5 *μ*L of 25 mM MgCl, 1 *μ*L dNTPs 10 mM, forward and reverse primer 0.5 *μ*L of a 100 pmol/*μ*L solution, 0.25 *μ*L Taq polymerase (GoTaq, Promega), and a final volume of 50 *μ*L with 5 *μ*L template DNA. Initial denaturation was done for 3 min at 95°C followed by 30 s at 95°C (denaturation), primer annealing for 30 s at 54°C, and extension for 1.30 min at 72°C. The cycle was repeated for 25 times and was followed by the final extension at 72°C for 5 min and held at 4°C. The PCR products were purified using the spinNaker gel and PCR DNA purification kit (Euroclone Spa, Italy). Sequencing of the purified PCR products was done using the sequencing 785F (forward primer)-5 GGA-TTA-GAT-ACC-CTG-GTA 3⁣′ and 907R (reverse primer) 5⁣′ CCG-TCA-ATT-CMT-TTR-AGT-TT3⁣′ (Eurofins Genomics, Germany) at Eurofins Genomics Germany GmbH.

## 3. Data Analysis

Antibacterial activity data was analyzed using SPSS Version 26_2019. Mean comparison was made using one-way ANOVA. Tukey's HSD was used for multiple pairwise comparisons, and *p* < 0.05 was considered statistically significant.

## 4. Results

### 4.1. Antibacterial Activity of the Endophytic Bacterial Crude Metabolites

Endophytic isolate Ch1 was isolated from *A. abyssinica*, En3, En5, and En6 from *K. petitiana*, Mk8 from *R. abyssinicus*, and Az10 from *C. longicauda*. The crude metabolites of the endophytic bacteria have shown a variable degree of inhibition against the test pathogens, with the inhibition zone ranging from the lowest value of 6.00 ± 0.00 mm against *P. aeruginosa* to the highest value of 22.00 ± 0.71 mm against *S.* Typhimurium. In general, *S.* Typhimurium was the most sensitive to the crude metabolites, followed by *E. coli*, and the least sensitive bacteria were *P. aeruginosa*. Isolate Ch1 has shown a higher degree of inhibition against *E. coli*, *S.* Typhimurium, and *P. aeruginosa*. On the other hand, *S. aureus* was the most sensitive to the crude metabolite of isolate En6 (Table 1).

### 4.2. MIC and MBC of Various Solvent Fractions of Metabolites From Endophytic Bacteria Against Human Pathogenic Bacterial Species

Selected solvent fractions of the endophytic bacterial metabolites resulted in significant antibacterial activity. The lowest MIC (0.03 ± 0.01) and MBC (0.98 ± 0.01) were recorded for the methanol–water fraction against *S.* Typhimurium. On the other hand, the highest MIC (125 ± 0.07) and MBC (250 ± 0.00) were recorded for the same fraction against *P. aeruginosa*, indicating its resistance to the metabolites (Table 2).

### 4.3. Molecular Identification

**Figure 3:**
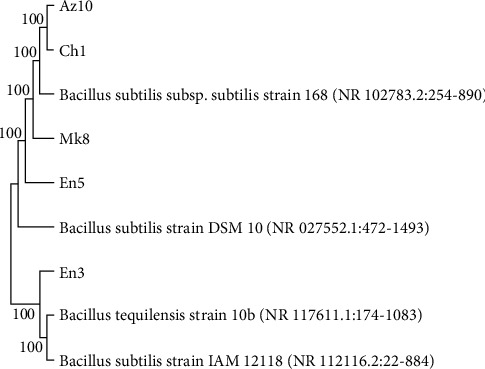
Phylogenetic tree of endophytic *B. subtilis* species identified using 16s rRNA sequence.

The 16S rRNA sequence analysis result confirmed that all of them belonged to the *B. subtilis* species except isolate En6 (Figure 3). However, the whole genome sequence analysis result of isolate En6 confirmed that it also belongs to the *B. subtilis* species, having 99.7% of percent identity.

## 5. Discussion

The antimicrobial assay of the selected endophytic metabolites resulted in broad-spectrum antibacterial activity against both Gram-positive bacteria *S. aureus* and three Gram-negative bacteria, including *P. aeruginosa*. This is the first work that reported the antibacterial activity of metabolites of endophytic bacteria isolated from the Ethiopian medicinal plants: *K. petitiana*, *A. abyssinica*, *R. abyssinicus*, and *C. longicauda.* Several previous reports revealed that the metabolites of these host medicinal plants have significant antimicrobial and other biological activities [25–30]. The crude metabolite of isolate En6 showed high activity against *S. aureus* compared to the rest. The metabolites, as a whole, exhibited lower activity against *P. aeruginosa* compared to the other test bacteria. *E. coli* and *S.* Typhimurium were more susceptible to all of the tested crude metabolites compared to *S. aureus* and *P. aeruginosa*, even though the isolate metabolites of the Ch1 resulted in the highest activity against most test bacteria (Table 1).

Molecular analysis confirmed that the endophytic bacterial isolates belong to the *B. subtilis* species. *B. subtilis* species are reported to produce more than 20 antibiotics with various chemical structures and commonly belong to the peptide and nonpeptide compounds [31]. Other researchers also reported the antimicrobial activity of metabolites of endophytic *B. subtilis* against human pathogens [16, 17]. Unlike our report, where the metabolites of the *B. subtilis* inhibited both Gram-negative and Gram-positive bacteria, selective antibacterial activity of metabolites from *B. subtilis* against Gram-negative bacteria was reported [32]. In another report, pure compounds isolated from *Bacillus* species, including *B. subtilis*, were reported to have high antibacterial activity against drug-resistant members of Gram-positive bacteria, especially *S. aureus*, but the metabolites in the report showed insignificant or no activity against Gram-negative bacteria [33]. Another study also reported significant antimicrobial activities of bacillibactin compounds (members of siderophores) produced by *B. subtilis* species against *Mycobacterium* species [34]. Unlike most siderophores that work by competition through iron chelating [11], bacillibactins are reported to have a direct antibiosis mechanism of action [35].

Various antibacterial substances and antibacterial mechanisms of metabolites produced by *B. subtilis* have been reported by different researchers. These include production of organic acids, alcohols, and esters [36] and secondary metabolites like ribosomal peptides (RPs), volatile compounds, polyketides (PKs), and nonribosomal peptides (NRPs) [34, 36]. The common antibacterial mechanisms of action of these antibiotics include inhibiting Gram-positive bacterial cell wall biosynthesis, particularly interfering with the synthesis of peptidoglycan by inhibiting the transportation of peptidoglycan precursors across the cell membrane, interfering with the functions of the cell membrane, as well as disrupting intracellular processes like DNA transcription, RNA translation, and protein metabolism [11, 37–39]. The PK antibiotic compounds bacillaene and macrolactins produced by *B. subtilis* are also among the reported antibiotics that work by selectively inhibiting prokaryotic protein synthesis [40, 41].

In the current work, even at the fraction level, the metabolites exhibited broad-spectrum activity against both the Gram-positive and Gram-negative human pathogenic bacteria, including *P. aeruginosa*, which makes the result highly promising. Novel catecholate, siderophore, bacillibactin compounds isolated from a strain of *B. subtilis* species were reported for their promising antimicrobial activity comparable to commercially available antibiotics against drug-resistant human bacterial pathogens [34]. Inhibition of siderophore biosynthesis, drug delivery, and iron starvation via competitive chelation are among the commonly described antimicrobial mechanisms of action of siderophores [42, 43]. Although further compound isolation, antibacterial assays, and analysis remained, the present study revealed that endophytic bacterial metabolites are the potential sources of antimicrobial agents.

The broad-spectrum activity of the metabolites observed also signifies the efficacy of the metabolites (Table 2). Based on the broad spectrum of significant antimicrobial activity of the fractions of the metabolites, the isolates are expected to be promising prospects for the discovery of new antibiotics that can be used as alternative antibiotic candidates against life-threatening human pathogenic bacteria.

## 6. Conclusion

In the current work, the endophytic *B. subtilis* isolated from the Ethiopian medicinal plants (*K. petitiana*, *A. abyssinica*, *R. abyssinicus*, and *C. longicauda*) was selected through continuous screening for its consistent antibacterial metabolite production and its broad-spectrum antibacterial activities and was identified at the molecular level using 16S rRNA gene sequence analysis. The metabolites of the selected isolates exhibited broad-spectrum antibacterial activity against human pathogenic bacteria. Selected fractions of the metabolites also resulted in high and broad-spectrum antibacterial activity. Generally, *P. aeruginosa* was found to be the most resistant to the metabolites of the endophytic bacteria compared to other pathogenic bacteria tested. Compound-level advanced works are required to confirm the various biological activities and chemical structures of the metabolites of the endophytic bacteria. In addition, the combined effects of the active metabolites and antibiotics need to be evaluated especially against the least sensitive test bacteria.

## Figures and Tables

**Table 1:** Inhibition zone of the crude metabolites of endophytic *B. subtilis* species against the bacterial pathogens.

**Isolate code**	**Zone of inhibition against the bacterial pathogens**			
	***S. aureus***	***E. coli***	***S.* Typhimurium**	***P. aeruginosa***
Az10	12.50 ± 0.71^b^	19.00 ± 1.41^ab^	20.00 ± 0.00^ab^	9.50 ± 0.71^a^
Ch1	11.50 ± 0.71^b^	21.00 ± 0.00^a^	22.00 ± 0.71^a^	10.00 ± 0.00^a^
En6	17.00 ± 1.41^a^	20.00 ± 0.00^ab^	20.000 ± 0.71^ab^	9.50 ± 0.71^a^
Mk8	9.00 ± 0.00^c^	19.50 ± 0.71^ab^	20.00 ± 0.00^ab^	7.00 ± 0.71^bc^
En3	10.50 ± 0.71^bc^	17.50 ± 0.71^ab^	17.50 ± 0.71^ab^	10.50 ± 0.71^a^
En5	10.50 ± 0.71^bc^	10.50 ± 0.71^b^	12.50 ± 0.71^bc^	6.00 ± 0.00^c^
Ciprofloxacin (10 *μ*g/mL)	12.50 ± 0.71	20.00 ± 0.00	21.00 ± 0.00	9.00 ± 0.00

*Note:* Az10, Ch1, Mk8, En5, En6, and En3 are codes of different endophytic *B. subtilis* isolates that produced the metabolites. The inhibition zones are presented as $\bar{x}\pm \text{SD}$. Values with different superscript letters within the same column are significantly different (*p* < 0.05).

**Table 2:** MIC and MBC of fractions from endophytic bacterial metabolites against human pathogenic bacteria.

**Isolate code**	**MIC and MBC of fractions from endophytic *B. subtilis* metabolites against human pathogenic bacteria ( ** $\bar{x}\pm SD$ **in mg/mL)**							
	***S. aureus***		***E. coli***		***S.* Typhimurium**		***P. aeruginosa***	
	**MIC**	**MBC**	**MIC**	**MBC**	**MIC**	**MBC**	**MIC**	**MBC**
Az10-Me-H_2_O 1:1	0.98 ± 0.00^a^	3.85 ± 0.07^a^	0.12 ± 0.00*a*	1.95 ± 0.01^a^	0.03 ± 0.01^a^	1.95 ± 0.01^b^	62.5 ± 0.071^b^	250 ± 0.000^c^
Ch1-Me-H_2_O 1:1	0.98 ± 0.00^a^	3.85 ± 0.01^a^	0.06 ± 0.00^a^	1.95 ± 0.01^a^	0.03 ± 0.01^a^	0.98 ± 0.01^a^	62.5 ± 0.07^b^	125 ± 0.071^b^
En6-Et-Me 1:1	0.98 ± 0.00^a^	3.85 ± 0.01^a^	0.06 ± 0.00^a^	3.89 ± 0.01^b^	0.06 ± 0.01^b^	7.88 ± 0.01^d^	31.25 ± 0.07^a^	62.5 ± 0.071^a^
Mk8-Me-H_2_O 1:1	3.85 ± 0.07^b^	15.63 ± 0.00^b^	0.12 ± 0.00^a^	3.89 ± 0.01^b^	0.03 ± 0.01^a^	3.85 ± 0.01^c^	NA	NA
En3-Me-H_2_O 1:1	0.98 ± 0.00^a^	15.63 ± 0.01^b^	0.12 ± 0.00^a^	7.81 ± 0.01^c^	0.04 ± 0.01^b^	3.85 ± 0.01^c^	125 ± 0.07^c^	125 ± 0.071^b^
En5-Me-H_2_O 1:1	3.85 ± 0.07^b^	62.5 ± 0.01^c^	0.93 ± 0.03^b^	15.63 ± 0.01^d^	1.98 ± 0.01^c^	15.63 ± 0.0^e^	125 ± 0.07^c^	NA

*Note:* Az10-Me-H_2_O 1:1: 50% methanol fraction; Et-Me 1:1: 50% methanol and 50% ethanol fraction; Az10, Ch1, Mk8, En5, En6, and En3 are codes of different endophytic *B. subtilis* isolates that produced the metabolites. The MIC (minimum inhibitory concentration) and MBC (minimum bactericidal concentration) are presented as $\bar{x}\pm \text{SD}$. Values with different superscript letters within the same column are significantly different (*p* < 0.05).

## Data Availability

The 16S rDNA sequences of each isolate identified in this study have been deposited in GenBank under Accession Numbers PQ535510, PV112413, PV126628, PV132993, PV132994, and PV132995 for the *B. subtilis* species. The data will be made available upon reasonable request.
